# MorphoGlia, an interactive method to identify and map microglia morphologies, demonstrates differences in hippocampal subregions of an Alzheimer’s disease mouse model

**DOI:** 10.3389/fncel.2024.1505048

**Published:** 2024-12-03

**Authors:** Juan Pablo Maya-Arteaga, Humberto Martínez-Orozco, Sofía Diaz-Cintra

**Affiliations:** Departamento de Neurobiología del Desarrollo y Neurofisiología, Instituto de Neurobiología, Santiago de Querétaro, Mexico

**Keywords:** machine learning, pipeline, hippocampus, clustering, image processing, UMAP

## Abstract

Microglia are dynamic central nervous system cells crucial for maintaining homeostasis and responding to neuroinflammation, as evidenced by their varied morphologies. Existing morphology analysis often fails to detect subtle variations within the full spectrum of microglial morphologies due to their reliance on predefined categories. Here, we present MorphoGlia, an interactive, user-friendly pipeline that objectively characterizes microglial morphologies. MorphoGlia employs a machine learning ensemble to select relevant morphological features of microglia cells, perform dimensionality reduction, cluster these features, and subsequently map the clustered cells back onto the tissue, providing a spatial context for the identified microglial morphologies. We applied this pipeline to compare the responses between saline solution (SS) and scopolamine (SCOP) groups in a SCOP-induced mouse model of Alzheimer’s disease, with a specific focus on the hippocampal subregions CA1 and Hilus. Next, we assessed microglial morphologies across four groups: SS-CA1, SCOP-CA1, SS-Hilus, and SCOP-Hilus. The results demonstrated that MorphoGlia effectively differentiated between SS and SCOP-treated groups, identifying distinct clusters of microglial morphologies commonly associated with pro-inflammatory states in the SCOP groups. Additionally, MorphoGlia enabled spatial mapping of these clusters, identifying the most affected hippocampal layers. This study highlights MorphoGlia’s capability to provide unbiased analysis and clustering of microglial morphological states, making it a valuable tool for exploring microglial heterogeneity and its implications for central nervous system pathologies.

## 1 Introduction

Since their discovery, microglia have intrigued researchers due to their ability to respond to external stimuli by changing morphology, migrating, and accumulating at lesion sites (Río-Hortega, 1919; Sierra et al., 2016). This responsiveness to different brain microenvironments demands a plastic behavior to perform a range of homeostatic processes, reflected in their varied morphologies (Sierra et al., 2014; Orihuela et al., 2016; Sierra et al., 2019; Matejuk and Ransohoff, 2020; Augusto-Oliveira et al., 2022; Lier et al., 2021; McNamara et al., 2023). Due to this versatility, microglia play a crucial role in understanding and addressing central nervous system disorders (Matejuk and Ransohoff, 2020; Li and Barres, 2018; Serrano-Reyes et al., 2022). Consequently, the quantitative assessment of microglial morphology has become widespread in neuroscience, particularly in evaluating neuroinflammatory states (De Biase and Bonci, 2019; Stratoulias et al., 2019; Green et al., 2022).

Understanding microglial morphology is relevant for detecting changes associated with their immediate response to local environmental signals (Stratoulias et al., 2019; Colombo et al., 2022; Reddaway et al., 2023). Researchers have developed tools for quantifying distinct morphological features and applied mathematical models to classify microglia (Stratoulias et al., 2019; Colombo et al., 2022; Reddaway et al., 2023). However, the diverse morphological states of these cells have resulted in an excessive number of nomenclatures. Common terms include “homeostatic” (resting, ramified), “reactive” (activated, inflammatory), “amoeboid” (phagocytic), and “rod” (Walker et al., 2013; Torres-Platas et al., 2014; Sierra et al., 2019; Felsky et al., 2019; Jurga et al., 2020; Guo et al., 2022; Reddaway et al., 2023). These classifications are limited, as they do not encompass the broad range of morphologies and thus overlook diverse functional aspects (Paolicelli et al., 2022). This inconsistency underscores the need for a methodology that embraces the continuous spectrum while categorizing morphological states (Stratoulias et al., 2019; Healy et al., 2022; Paolicelli et al., 2022; Vidal-Itriago et al., 2022; Reddaway et al., 2023; Green and Rowe, 2024).

The best approach for characterizing microglial morphology is still debated (Green et al., 2022; Reddaway et al., 2023; Green and Rowe, 2024). Common methods for analyzing microglial morphology include skeleton analysis, cell body area and perimeter, fractal analysis, and Sholl analysis (Green et al., 2022). Manual methods and neural networks have faced challenges due to the requirement of *a priori* labeling by an expert, which can miss subtle changes in the continuous spectrum of microglial morphologies. Machine learning methods have often been limited to focusing on just one characteristic of microglia, typically their branching. To capture the full morphological variability, it is necessary to quantify multiple microglial features (Colombo et al., 2022; Paolicelli et al., 2022; Reddaway et al., 2023; Green and Rowe, 2024). Additionally, there is controversy on whether to analyze entire images or focus on single-cell analysis (Green et al., 2022). Full photomicrograph analysis may introduce biases and reduce effect sizes, whereas single-cell analysis, though more labor-intensive, often reveals greater statistical differences but may provide a biased sample due to selective cell inclusion (Green et al., 2022).

According to a consensus, while naming and categorizing are useful, these constructs are artificial because biological phenomena exist on a spectrum (Paolicelli et al., 2022). Considering this and the complexity of microglial states, we have developed a computational pipeline called MorphoGlia that addresses both aspects. This pipeline captures the complexity of microglia by displaying their spectrum of morphological states while enabling their neutral categorization in an unbiased manner based on reproducible morphology measurements.

MorphoGlia provides a user-friendly interface designed to extract microglial morphological features and apply advanced machine learning techniques to identify those most effective in distinguishing between study groups. These selected features are projected into a common space and clustered, enabling a detailed examination of distinct group-specific patterns. From this projection, each data point represents an individual microglial morphology, which is then mapped and color-coded by its cluster back onto the original tissue photomicrograph. This functionality allows users to validate the morphological states identified by the machine learning ensemble and analyze the spatial concentration of different morphologies, aiding in the identification of the most affected regions.

This comprehensive approach can be applied to studying pathologies where microglia play a significant role. In Alzheimer’s disease (AD), brain cells undergo morphological changes as the pathology progresses, making the study of microglial morphology crucial for understanding the neuroinflammatory processes involved (Abdelghany et al., 2022). To this end, MorphoGlia was tested to evaluate microglial morphology in the hippocampus of a mouse model of Alzheimer’s-like disease induced by scopolamine (SCOP). The administration of this muscarinic receptor antagonist mimics both the behavioral (cognitive impairment) and molecular (acetylcholine deficiency, neuroinflammation) features of AD (Klinkenberg and Blokland, 2010; Karthivashan et al., 2018; Chen and Yeong, 2020). Nonetheless, the microglial morphology in this model has not been investigated. This study demonstrates that MorphoGlia provides a user-friendly and unbiased method to differentiate between various microglial morphology states.

## 2 Materials and methods

### 2.1 Experimental animals

All experimental procedures with animals were carried out in accordance with National Institute of Health Guide for Care and Use of Laboratory Animals and the project was approved by the Bioethics Committee of the Instituto de Neurobiología (INB) of the Universidad Nacional Autónoma de México (UNAM) (registration number 117). Male C57BL/6 mice, aged 10–12 weeks and with an initial body weight ranging from 20 to 30 g, were used for this study. The animals were sourced from the vivarium facility of the Institute of Neurobiology. They were housed in polycarbonate cages, with 2–3 mice per cage, and provided with water and chow (Purina^®^ 5001) *ad libitum*. The housing conditions were controlled, maintaining a temperature of 21–25°C, relative humidity of 45–65%, and a 12-h inverted dark/light cycle.

### 2.2 Pharmacological treatment

A solution of scopolamine hydrobromide trihydrate (SCOP, Sigma-Aldrich, #S0929) was prepared in sterile 0.9% saline solution (SS). The mice were divided into two groups: a control group (*n* = 4), which received SS (vehicle), and an experimental group (*n* = 4), which received 1.0 mg/kg SCOP. Both the SS and SCOP solutions were administered daily via intraperitoneal injection during the morning period (9:00–10:00 a.m.) for nineteen consecutive days.

### 2.3 Tissue preparation

Mice were euthanized by intraperitoneal injection of sodium pentobarbital (250 mg/kg). After anesthesia, the animals were intracardially perfused with 4% p-formaldehyde (PFA) in 0.1 M phosphate buffer saline pH 7.4 (PBS). Brains were collected, post-fixed in PFA for 48 h at 4°C, and cryoprotected in 35% sucrose in PBS for 7 days at 4°C. The brains were then bisected, and the right hemisphere was used to obtain 40 μm sagittal sections in a cryostat. Slices were stored in PBS with 0.1% sodium azide at 4°C until analysis.

### 2.4 Enzymatic immunohistochemistry

Microglia were stained with antibodies against Iba-1 protein and an antibody signal enhancer to address over-fixation and non-specific binding (Rosas-Arellano et al., 2016). For analysis, 8–10 slices from the dorsal hippocampus (lateral 0.48 – 1.90 mm) were selected. Sections underwent antigen retrieval in citrate buffer (pH 6.0) at 80°C for 20 min, quenching in 3% H2O2 and 10% methanol in PBS for 30 min, and enhancement in 0.1% Triton X-100, 0.5% Tween-20 in PBS with 50 mM glycine for 45 min. They were incubated overnight at 4°C with anti-Iba-1 antibody (1:500, Abcam, #ab107159). The next day, sections were washed, incubated with secondary biotinylated antibodies (1:500, Vector Laboratories, #BA-9200) for 2 h, and processed with the VECTASTAIN^®^ ABC-HRP Kit (Vector Laboratories, #PK-4000). The signal was developed with a solution containing 0.0005 g/ml diaminobenzidine, 0.03 g/ml nickel ammonium sulfate, 0.006 g/ml dextrose, 0.0012 g/ml NH4Cl, and 0.0001 mg/ml glucose oxidase until the color changed to purple. The reaction was then stopped with water, and sections were stored in PBS at 4–8°C for 24 h. Finally, sections were mounted, air-dried, dehydrated, cleared in xylene, and covered with Entellan^®^ before being sealed with coverslips for image acquisition.

### 2.5 Image acquisition

Digital images of Iba-1 + cells in the CA1 and dentate gyrus subregions of the hippocampus were captured using a Nikon Eclipse Ci-Li light microscope equipped with a Nikon Plan 40 × /0.65 air lens. Photomicrographs were taken from four equidistant slices per brain (*n* = 4).

### 2.6 Image pre-processing

Digital images were pre-processed under blinded conditions in Image J Fiji software based on a previous protocol (Young and Morrison, 2018; Supplementary Figures 4A, B). Briefly, each image was converted into an 8-bit image, and a lookup table of grays was applied. After that, brightness and contrast were adjusted using the Auto function, and an unsharp mask (Radius sigma = 3.0 pixels) and noise elimination (despeckle) were applied to the image. The threshold function was used for segmentation, and each Iba-1 + cell in the image was completely reconstructed manually using drawing tools (Pencil tool, size = 2.0) to finally obtain binary images. Afterward, binary images were noise-smoothed by applying despeckle, close (option for binary), and remove outliers (default options); cleaned by erasing all the background marks; and finally saved in .tiff format for further processing.

### 2.7 MorphoGlia code, interface and software

MorphoGlia has been developed with a focus on user-friendliness and accessibility, making it an ideal tool for the broader scientific community. The software is available in two main modes: a software mode and an interface mode, both designed to facilitate ease of use. The executable file was generated using PyInstaller,^1^ while the interactive interface was built using the Tkinter Python library.^2^ For advanced users, direct modification of the source code is recommended to tailor the application to the specific needs of individual experiments. This approach allows for greater flexibility and customization, ensuring that MorphoGlia can be adapted to a wide range of research scenarios. All components of MorphoGlia, including the executable file, interface mode, and code mode, are available for download from the following GitHub repository: https://github.com/Maya-Arteaga/MorphoGlia. Currently, the executable file is limited to use on macOS with M1/M2 processors.

Please refer to the video tutorial attached in supplementary information section for a practical demonstration of how to utilize the MorphoGlia software (Supplementary Video 1).

### 2.8 Morphology analysis

Each cell was identified in the complete binary photomicrographs, and classic morphometric features were computed using Python, primarily with the OpenCV library.^3^ For skeleton analysis, the total branch length (in pixels), number of initial points (cell processes emerging from the soma), number of junction points (branch subdivisions), and number of endpoints (ends of branches) were measured. Cell body analysis included calculating the area, perimeter, circularity (with 1 representing a perfect circle), Feret diameter (maximum caliper diameter), compactness (how closely an object packs its area), aspect ratio (width/height), orientation (angle in degrees), and eccentricity (major axis/minor axis). The same metrics used in cell body analysis were applied to the entire cell. Fractal analysis involved determining convex hulls (the smallest convex set of pixels enclosing a cell) and performing the same calculations as in cell body analysis, as well as calculating the fractal dimension. Sholl analysis consisted of identifying the number of Sholl circles (circles with increasing radii created around the centroid of the cell soma), counting crossing processes (intersections of cell processes with Sholl circles), and measuring the maximum distance (distance between the centroid and the four vertices of the image). These metrics allow researchers to analyze the biologically relevant characteristics of the cell. During morphometric data extraction, a directory is created for each image containing the segmented cells and corresponding analyses. This approach ensures rigorous quality control.

### 2.9 Feature selection

Selecting the most appropriate features to characterize microglia is challenging due to significant biological variability depending on the region and pathology. A fixed set of features that best differentiates morphological states cannot be universally applied. To address this, a dynamic feature selection approach is necessary to ensure relevance and mitigate noise. We employed the Recursive Feature Elimination (RFE) algorithm, a specialized technique for selecting crucial features by iteratively reducing the feature set and removing the least important ones. RFE uses a Random Forest engine as the underlying training model to determine feature importance. The Random Forest algorithm ensures robustness, avoids overfitting, and captures non-linear relationships between features and the target variable. In this study, the groups (SS-CA1, SCOP-CA1, SS-Hilus, and SCOP-Hilus) were used as the target variable. The features analyzed were those computed in the morphology analysis, totaling 32 variables. The RFE algorithm selected the most significant half of these features, enhancing the robustness of the model and enabling feature selection suited to the specificities of each study group. This approach was implemented using the scikit-learn package.^4^

### 2.10 Dimensionality reduction

Uniform Manifold Approximation and Projection (UMAP) is a technique designed for non-linear and non-parametric dimensionality reduction. This technique preserves both local and global structures of the data. It assumes that the data is uniformly distributed on a Riemannian manifold with a locally constant Riemannian metric and local connectivity (Taylor et al., 2014; Becht et al., 2018). Key UMAP parameters include the number of nearest neighbors (n_neighbors) and the minimum distance between points (min_dist). The n_neighbors parameter constructs the high-dimensional neighborhood graph, with lower values focusing on local structures and higher values capturing global structures. Recommended values range from 5 to 50. The min_dist parameter controls point clustering, with lower values resulting in tighter clustering and higher values in more dispersed points. The recommended min_dist value is 0.1. Additionally, the number of components (n_components) determines the dimensionality for data reduction. UMAP uses fuzzy set theory to represent the probability distribution in both high-dimensional and low-dimensional spaces, preserving complex data patterns through non-linear embedding (Taylor et al., 2014; Becht et al., 2018). This makes UMAP effective for capturing intricate relationships in the data. This approach was implemented using the scikit-learn package.^5^ For further details consult https://github.com/lmcinnes/umap.

It is essential to adjust UMAP hyperparameters (n_neighbors and min_dist) based on the data characteristics and experimental goals. Various hyperparameters were tested, demonstrating robust results (Supplementary Figure 1).

### 2.11 Clustering

Following UMAP for dimensionality reduction, Hierarchical Density-Based Spatial Clustering of Applications with Noise (HDBSCAN) was used for clustering. This non-parametric method constructs a cluster hierarchy based on the multivariate modes of the underlying distribution by transforming the space according to density, building the cluster hierarchy, and extracting the clusters. HDBSCAN’s density-based approach makes minimal assumptions about the clusters, identifying them as regions of high density separated by low-density regions, thus eliminating the need to specify the number of clusters beforehand. This method can identify clusters of varying shapes and sizes and creates a hierarchy based on different density levels. Key hyperparameters include the minimum cluster size (min_cluster_size) and minimum cluster samples (min_samples). The minimum cluster size determines the smallest number of points in a cluster for it to be considered valid; fewer points are treated as noise. Min_samples defines the minimum number of neighboring points required for a point to be considered a core point, which must include at least the specified number of sample points (including itself) in its neighborhood. HDBSCAN effectively handles data noise by excluding points that do not fall within high-density regions, making it robust to noise and outliers. This approach was implemented using the scikit-learn package.^6^ For further details consult https://github.com/scikit-learn-contrib/hdbscan.

### 2.12 Spatial analysis and visualization

Dimensionality reduction and clustering result in color-coded data points, each representing a cell. These color-coded cells are mapped back onto the tissue microphotograph to visualize their spatial arrangement (Figures 5A, B and Supplementary Figures 4C, D). This approach serves two main purposes: confirming similarities among cells within the same cluster and providing insights into the spatial distribution of each clustered cell. This visualization is particularly useful for spatial analyses, allowing for the identification of the most affected zones and uncovering previously unexplored patterns in disease physiopathology. In the original images, color-coded microglia were assessed within the CA1 hippocampal layers: stratum oriens (SO), stratum pyramidale (SPyr), and stratum radiatum (SR). The number of each cluster-type microglia was then estimated by manual cell counting in the different zones of the CA1 subregions for both the SS and SCOP groups. Additionally, we utilized the Allen Brain Explorer^®^ Beta viewer^7^ to obtain spatial references of the CA1 region from frontal and superior perspectives. This tool facilitated enhanced spatial context, enabling precise localization of the slice with the greatest lateral impact.

### 2.13 Statistical analysis

Statistical analysis was performed using R (v4.4.0). Correlation plots and chi-square tests were generated using the corrplot and gplots libraries, respectively, while heatmaps were created with the ComplexHeatmap library. The chi-square test determined significant associations between study groups (SS-CA1, SCOP-CA1, SS-Hilus, SCOP-Hilus) and clusters from the UMAP-HDBSCAN analysis (Clusters 0–4). Standardized residuals were calculated to identify the nature of these associations, with positive values indicating higher observed frequencies and negative values indicating lower observed frequencies compared to expected values. This test assesses the independence of categorical variables, and standardized residuals highlight specific associations contributing to the overall chi-square statistic, helping to link morphological states (clusters) with study groups. Heatmaps were used to visualize variable correlations with study groups or clusters, facilitating rapid condition comparisons. Spatial analysis and visualization data were analyzed using GraphPad Prism 8 (GraphPad Software, Inc.). The Mann–Whitney U test was performed to evaluate differences in the number of cells and relative frequency per cluster for each CA1 layer of each image, with a *p*-value < 0.05 considered significant. The sample size was calculated using the following parameters: P(X > Y): 0.8; Alpha two-sided: 0.05; Power: 0.8.

## 3 Results

### 3.1 MorphoGlia is a user-friendly interface that receives user inputs according to specific research requirement

**FIGURE 1:**
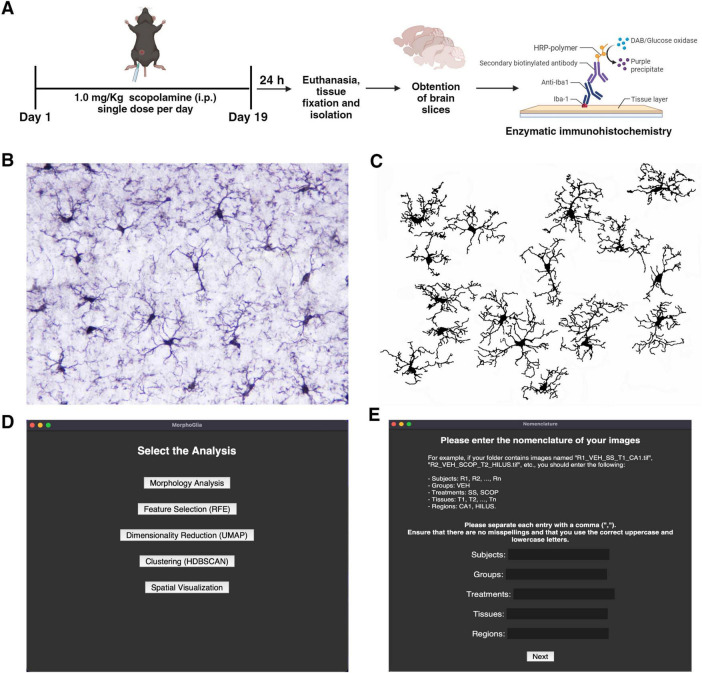
Scopolamine protocol, image pre-processing and MorphoGlia interactive tool. **(A)** SCOP treatment protocol in mice and enzymatic immunohistochemistry (image created in BioRender.com). **(B)** Representative image of the hippocampus after Iba-1 immunostaining. **(C)** Binary image of Iba-1 + cells processed using Image J Fiji. **(D)** MorphoGlia allows users, to select analyses with a simple click. **(E)** MorphoGlia is designed to be a user-friendly tool, enabling users to input parameters tailored to their specific experiments. SCOP, scopolamine.

In this study, we evaluated microglial morphology in the hippocampus of C57BL/6 mice following chronic treatment with 1.0 mg/kg (i.p) SCOP (Figure 1A). To visualize microglia in the hippocampal subregions, we performed enzymatic immunohistochemistry using specific antibodies against Iba-1 (Figure 1B). We captured four images of each subregion, CA1 and Hilus, for both the SS and SCOP groups, resulting in a total of 64 images (SS-CA1 = 16; SCOP-CA1 = 16; SS-Hilus = 16; SCOP-Hilus = 16). These photomicrographs were preprocessed to obtain binarized images (Figure 1C).

Once the binary images are prepared, the MorphoGlia interface (Figures 1D, E) or code mode can be used to execute our proposed pipeline. This pipeline involves five steps (Figure 1D): (1) morphology analysis, which extracts 32 microglial morphology features; (2) feature selection, where the RFE algorithm selects the 16 best features that distinguish the study groups; (3) dimensionality reduction, which employs UMAP to project the 16 selected features into a two-dimensional space; (4) clustering, which uses HDBSCAN, a non-supervised algorithm with noise detection, providing a more objective clustering method; and finally, (5) spatial visualization, where cells are color-coded according to their cluster and mapped back onto the tissue microphotograph to visualize their spatial arrangement, facilitating the identification of the most affected zones.

### 3.2 MorphoGlia analyses multiple morphometric features and identifies the most significant features for morphological differentiation

**FIGURE 2:**
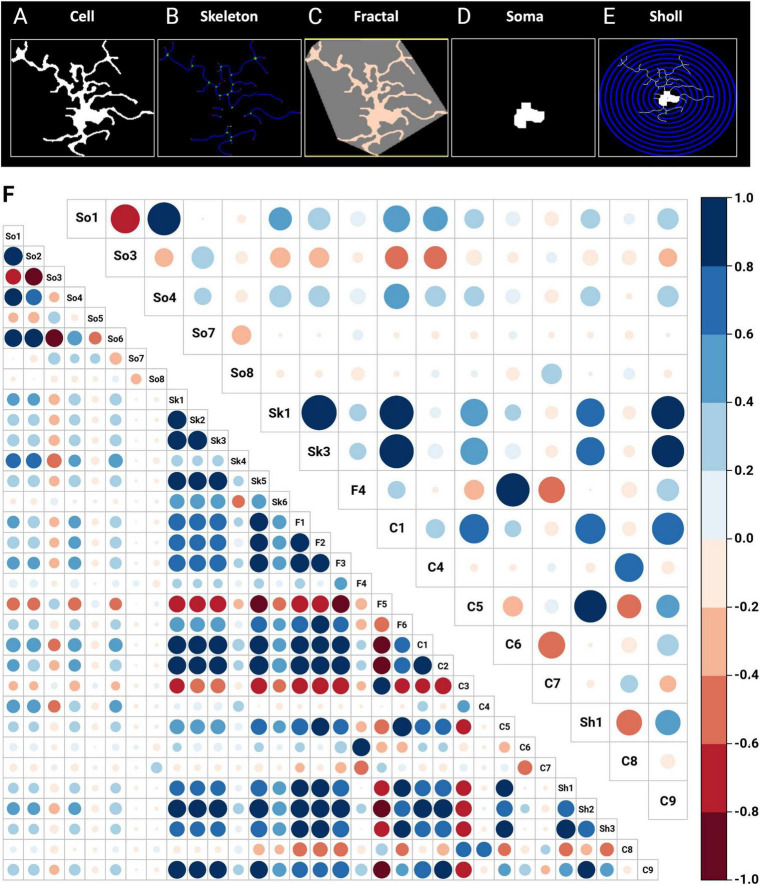
Morphology analysis and feature selection via RFE. In the morphological analysis, 32 features are initially extracted through automated data processing using **(A)** cell, **(B)** skeleton, **(C)** fractal (convex hull), **(D)** soma and **(E)** Sholl features. **(F)** In the feature selection process, RFE with a random forest algorithm identified 16 key features as the most significant for distinguishing between the studied groups: SS-CA1, SCOP-CA1, SS-Hilus, and SCOP-Hilus. The bottom left corner shows the correlation matrix of the 32 initially extracted features, while the top right corner displays the correlation matrix of the 16 features selected by RFE. The size and color of the circles indicate the strength of the correlations, with blue representing positive correlations and red representing negative correlations. C, cell; F, fractal (convex hull); RFE, recursive feature elimination; SCOP, scopolamine; Sh, Sholl; Sk, skeleton; So, soma; SS, saline.

From the 64 images, a total of 1,221 cells were identified, with an average of 19.09 cells per image (Supplementary Table 1). MorphoGlia’s Morphology Analysis extracted 32 features (Supplementary Table 2) from each cell based on five types of analyses: (1) Skeleton analysis, (2) Soma analysis, (3) Cell analysis, (4) Fractal (Convex Hull) analysis, and (5) Sholl analysis (Figures 2A–E).

Once the features were obtained (Figure 2F, bottom left corner correlation matrix), to address the significant heterogeneity in microglia morphology depending on the region and pathology, we used RFE with a Random Forest engine to select the most significant variables that distinguished our study groups (SS-CA1, SCOP-CA1, SS-Hilus, and SCOP-Hilus) as the target variable. This step reduces noise and enhances the performance of dimensionality reduction algorithms (Figure 2F, top right corner correlation matrix).

### 3.3 MorphoGlia detects morphology clusters without prior categorization

**FIGURE 3:**
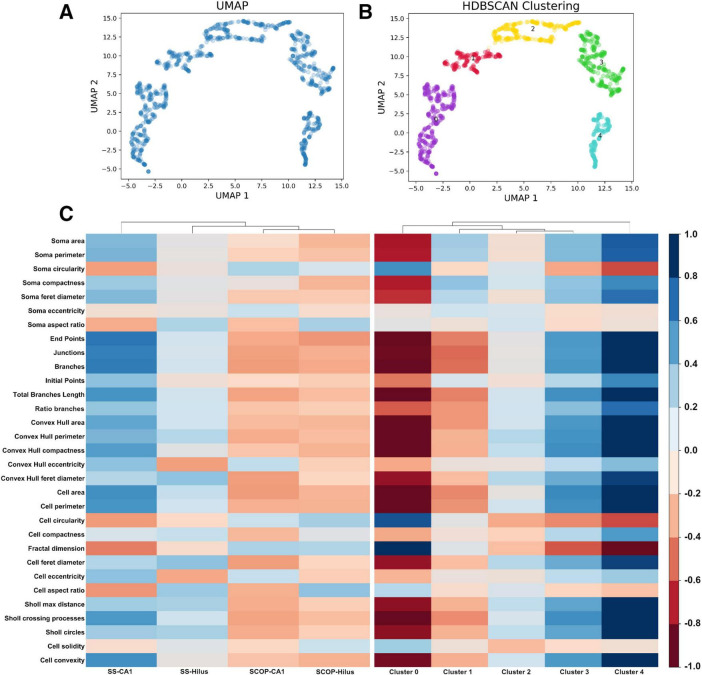
Uniform Manifold Approximation and Projection dimensionality reduction and HDBSCAN clustering. **(A)** UMAP dimensionality reduction in a two-dimensional space using the 16 features selected by RFE. **(B)** HDBSCAN clustering identifies five distinct color-coded clusters. **(C)** Heatmaps comparing the study groups and the resulting clusters using the 32 extracted features from the morphological analysis: (Left) the study groups exhibit less variance in morphology features, indicating an overall similarity in the photomicrograph’s cell features, which masks the subtle details of individual cells. (Right) The resulting clusters show increased variance in the morphology features, suggesting that clustering reveals hidden patterns, effectively highlighting the importance of the clustering process. HDBSCAN, Hierarchical Density-Based Spatial Clustering of Applications with Noise; UMAP, Uniform Manifold Approximation and Projection.

Once the most significant features were obtained, to reduce their dimensionality, we applied a non-linear dimensionality reduction technique called UMAP (Figure 3A), which converted the 16 features into two dimensions (n_components = 2). Notably, despite varying hyperparameters, the data structure was preserved (Supplementary Figure 1). The hyperparameters that best preserved both global and local structures were n_neighbors = 10 and min_dist = 0.1.

Subsequently, to perform an unbiased classification, we employed HDBSCAN clustering with parameters set to a minimum cluster size of 20 and a minimum sample size of 10. This analysis resulted in 5 distinct clusters (Figure 3B and Supplementary Table 1) and identified three cells as not belonging to any cluster (Supplementary Figure 2), leading to a total of 1,218 cells classified. To determine if our pipeline identified a pattern of microglial morphology in the obtained clusters, we created heatmaps contrasting the variables with the study groups and the obtained clusters (Figure 3C). Additionally, we generated confusion matrices targeting the study groups and the obtained clusters to evaluate whether clustering improved the algorithm’s performance by avoiding the mixing of morphological states within the study groups (Supplementary Figure 3).

### 3.4 MorphoGlia effectively differentiates between the study groups and identifies morphologies strongly associated with health and pathology

**FIGURE 4:**
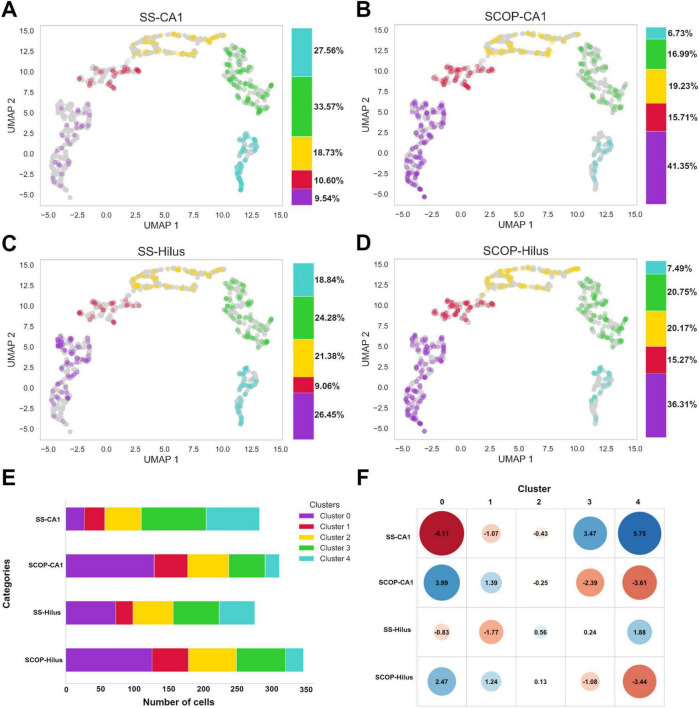
Study group distributions across clusters, cell count and standardized residuals. Scatter plots of panel **(A)** SS-CA1, **(B)** SCOP-CA1, **(C)** SS-Hilus, and **(D)** SCOP-Hilus groups across the manifold and obtained clusters. The color bar indicates the cluster percentages of cells in each cluster for the study groups. **(E)** Cluster cell count shows the absolute number of cells in each cluster for the study groups. Notice that the SCOP groups have a higher cell count compared to the SS groups. **(F)** Standardized Residuals of the clusters in the study groups present the standardized residuals for each cluster within the study groups. Clusters 0 and 1 exhibit significant negative associations with the SS groups (red), while Clusters 3 and 4 are positively associated with the SS groups (blue). Notably, the SCOP groups display the opposite association pattern. SCOP, scopolamine; SS, saline.

First, we evaluated the distribution of the study groups within the manifold and obtained clusters, as well as the percentage of each cluster in the study groups (Figures 4A–D). We then quantified the total number of cells identified in each group (Figure 4E) and calculated the mean number of cells per image: SS-CA1 had 283 total cells (mean: 17.68 cells per image), SCOP-CA1 had 312 total cells (mean: 19.50 cells per image), SS-Hilus had 276 total cells (mean: 17.25 cells per image), and SCOP-Hilus had 347 total cells (mean: 21.68 cells per image) (Figure 3A, Supplementary Figure 5, and Supplementary Table 1).

Given the observed variations in the number of cells corresponding to each cell group among the study groups, we investigated the relationship between the obtained clusters and the study groups using a chi-square test. The test yielded a chi-square statistic of 148.84, *p* < 2.2e−16, indicating a significant relationship between the clusters and the study groups. To determine which clusters were most associated with each study group, we calculated standardized residuals. Clusters 0 and 1 exhibited significant negative associations with the SS groups (marked in red), suggesting that these clusters are less prevalent in the SS groups. In contrast, Clusters 3 and 4 were positively associated with the SS groups (marked in blue), indicating a higher prevalence of these clusters in the SS groups. The SCOP groups displayed the inverse pattern, with positive associations in Clusters 0 and 1 and negative associations in Clusters 3 and 4 (Figure 4F).

### 3.5 MorphoGlia allows users to delimit the most affected CA1 hippocampal distances and layers by pathology

**FIGURE 5:**
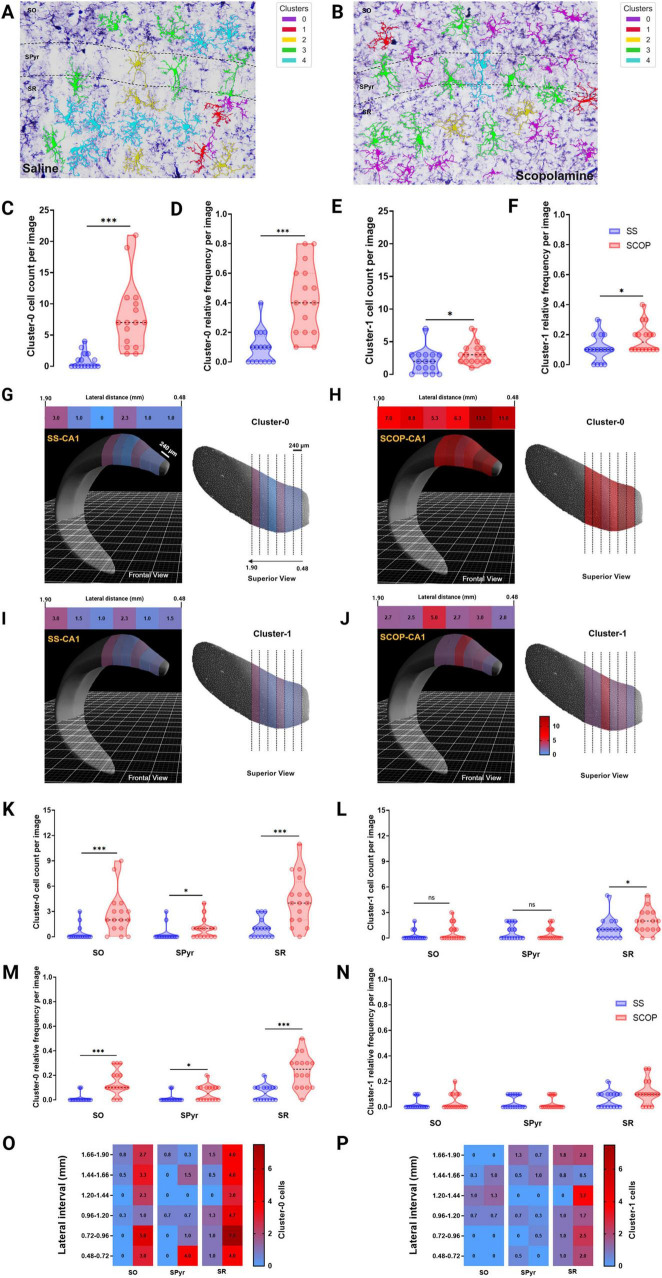
MorphoGlia spatial visualization and distance and spatial analysis. **(A,B)** Representative 40× photomicrographs of the hippocampal CA1 subregion and its sublayers of saline **(A)** and scopolamine **(B)** groups, showing immunostained Iba-1 positive cells mapped by MorphoGlia. **(C,E)** violin plots of microglia cluster cell count: violin plots displaying the microglia cell count for panel **(C)** Cluster 0 and **(E)** Cluster 1 within the CA1 region of both the saline (SS) and scopolamine (SCOP) groups. **(D,F)** Violin plots of microglia cluster relative frequency: violin plots displaying the microglia relative frequencies for panel **(D)** Cluster 0 and **(F)** Cluster 1 within the CA1 region of both the SS and SCOP groups. **(G,J)** The Allen Brain Explorer^®^ Beta 3D viewer was utilized to accurately localize lateral distances within the CA1 region for Cluster 0 cells of the SS group (**G**, frontal view in the left; superior view in the right) and SCOP group (**H**, frontal view in the left; superior view in the right), as well as for Cluster 1 cells of the SS group (**I**, frontal view in the left; superior view in the right) and SCOP group (**J**, frontal view in the left; superior view in the right). Heatmap upper image are presented for each group and indicate mean cell count of panels **(G,H)** Cluster 0 and **(I,J)** Cluster 1 cells at varying lateral distances (0.48–1.90 mm, in 240 μm intervals) along the CA1 region of the dorsal hippocampus. **(K,L)** The violin plots indicate the cell count of panel **(O)** Cluster 0 and **(P)** Cluster 1 microglia per image in the *Stratum radiatum* (SR), *Stratum oriens* (SO), and *Stratum pyramidale* (SPyr) sublayers of the dorsal hippocampus. **(M,N)** The violin plots indicate the relative frequency of panel **(O)** Cluster 0 and **(P)** Cluster 1 microglia per image in the stratum SR, SO and SPyr sublayers of the dorsal hippocampus. **(O,P)** The heatmaps depict the mean cell count of panel **(O)** Cluster 0 and **(P)** Cluster 1 microglia in the CA1 sublayers of the dorsal hippocampus (lateral distance 0.48–1.90 mm, 240 μm intervals). The Mann–Whitney U test was performed to evaluate differences in the number of cells per cluster. Significant differences are indicated by asterisks (*p < 0.05, ***p < 0.001).

To investigate the spatial distribution of cells based on their cluster labels, we mapped the cells back onto the tissue microphotograph to visualize their arrangement (Figures 5A, B). To validate the strong associations of Cluster 0 (3.99 and 2.47) and the moderate associations of Cluster 1 (1.39 and 1.24) with the SCOP groups (Figure 4F), we examined their number and relative frequencies within the CA1 region. Our analysis confirmed a significant increase in both number and relative frequency of Cluster 0 (*p* < 0.001) and Cluster 1 (*p* < 0.05) cells in the CA1 region of SCOP-treated mice (Figures 5C–E).

Following this confirmation, we analyzed the cell count distribution of these clusters across different lateral coordinates (from 0.48 to 1.90 mm in 240 μm intervals) to identify specific locations with elevated cluster concentrations (Figures 5G–J). This analysis revealed a marked increase in Cluster 0 cells in the 0.48–0.96 mm interval (Figures 5G, H) and an increase in Cluster 1 cells in the 1.20–1.44 mm interval (Figures 5I, J) in the dorsal CA1 of SCOP treated mice compared with SS group.

Finally, to determine whether these clusters were predominantly located within specific layers of the CA1 region of the hippocampus, we further divided the CA1 subregion into its anatomical layers: SO, SPyr and SR (Figures 5A, B). We then quantified the number and relative frequencies of these clusters within each layer as observed in the microphotographs (Figures 5K–P). This analysis showed that Cluster 0 was significantly more prevalent in SO (*p* < 0.001), SPyr (*p* < 0.05), and SR (*p* < 0.001) of SCOP mice compared with SS group (Figures 5K, M). Conversely, number of Cluster 1 cells was significantly higher only in SR (*p* = 0.040), with no significant differences observed in SO (*p* = 0.265) or SPyr (*p* = 0.811) between the SCOP and SS groups (Figure 5L). However, no significant differences were found among groups for the relative frequencies of Cluster 1 cells of any hippocampal sublayers (Figure 5N). Therefore, the distribution analysis across different lateral coordinates for this data (Figures 5O, P) revealed that the specific locations of SR and SO with higher number of Cluster 0 cells are consistent with those found in the dorsal CA1 for SCOP animals.

## 4 Discussion

**FIGURE 6:**
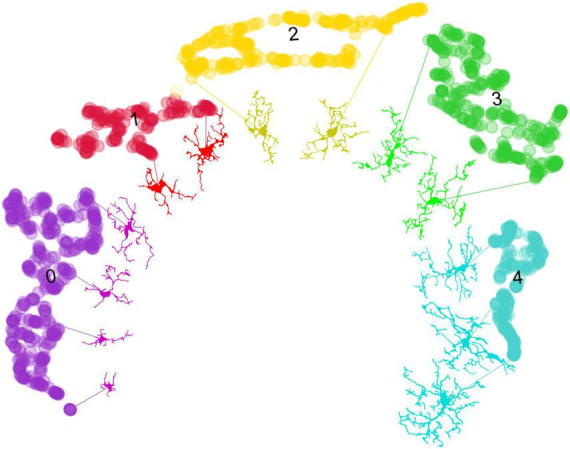
From data points to microglial morphologies: visualizing cluster variability. This figure demonstrates the application of the umap.plot.interactive() function to trace individual microglial cells to their respective data points within the UMAP space. Despite the effective clustering of morphological states by HDBSCAN, there is variability within the clusters. This variability arises from the 16 selected features, which have been reduced to two dimensions through UMAP, capturing a broad spectrum of microglial morphologies. Notably, MorphoGlia displays these morphologies along a curved continuum, illustrating a gradual transition across diverse microglial states. This visualization underscores the complexity of microglial morphology and the utility of advanced clustering techniques in identifying subtle differences within grouped data.

In this study, we demonstrated the application of MorphoGlia for identifying microglial morphology clusters. MorphoGlia effectively captures the full range of microglial morphologies (Figure 6), highlighting a continuous transition across various morphological states, while also facilitating clustering and delineating the most affected regions (Figures 3–6). This approach not only aligns with current research trends by embracing the concept of a morphological continuum and detecting subtle variations, but it also identifies clusters of morphological states that correspond to traditional classifications described in the literature.

The morphological state commonly referred to as “amoeboid” is typically found in neuroinflammatory microenvironments (Parakalan et al., 2012; Reddaway et al., 2023; Jurga et al., 2020; Masuda et al., 2020; Leyh et al., 2021). This state is morphologically characterized by retracted processes and a more circular shape, facilitating migration to the injury site (Parakalan et al., 2012; Reddaway et al., 2023; Savage et al., 2019; Jurga et al., 2020; Masuda et al., 2020; Leyh et al., 2021; Green and Rowe, 2024). This description corresponds to Cluster 0, which stands out for having greater circularity and cell solidity compared to other clusters (Figure 3C, heatmap). On the other hand, the morphological state known as “reactive” is associated with neuronal damage, inflammatory cytokine production, phagocytosis, and migration to injury sites (Tynan et al., 2010; Ziebell et al., 2015; Reddaway et al., 2023; Savage et al., 2019; Leyh et al., 2021). This state is morphologically characterized by having fewer and thinner branches (Tynan et al., 2010; Ziebell et al., 2015; Savage et al., 2019; Leyh et al., 2021; Colombo et al., 2022). This description aligns with Cluster 1 (Figures 3B, C, 6). These two morphological clusters are more associated with the SCOP groups than with the SS groups (Figure 4F), which is consistent with their described functionalities.

Other commonly recognized morphological states are “homeostatic” and “hyper-ramified.” Healthy brain microglia are in a “homeostatic” state and search for signs of infection or distress (Glenn et al., 1992; Reddaway et al., 2023; Leyh et al., 2021). Homeostatic microglia are morphologically characterized by elongated and branched processes (Glenn et al., 1992; Felsky et al., 2019; Leyh et al., 2021; Green and Rowe, 2024). This description corresponds to Clusters 3 and 4 (Figures 3B, C, 6), which are more strongly associated with the SS groups than with the SCOP groups (Figure 4F). Furthermore, the termed “hyper-ramified” appears upon detection of a noxious stimulus, serving as a transitional state between the homeostatic and reactive states (Glenn et al., 1992; Reddaway et al., 2023; Leyh et al., 2021). However, it has also been described in response to non-pathological stimuli (Torres-Platas et al., 2014). This state is morphologically characterized by having more complex processes than the homeostatic state (Glenn et al., 1992; Leyh et al., 2021; Colombo et al., 2022). This description might correspond to Cluster 4 (Figure 3C, heatmap); however, it is more associated with the SS groups.

The morphological state often referred to as “rod” is usually juxtaposed with neuronal elements to facilitate their repair or breakdown (Glenn et al., 1992; Taylor et al., 2014; Reddaway et al., 2023; Holloway et al., 2019). However, their role in disease is not well characterized (Parakalan et al., 2012). This state is morphologically characterized by being bipolar with a thin and elongated soma (Taylor et al., 2014; Reddaway et al., 2023; Holloway et al., 2019; Savage et al., 2019; Giordano et al., 2021; Green and Rowe, 2024). This morphological state seems to be present but not clearly defined in a specific cluster. It is shared by Clusters 2 and 3, depending on the level of branching. Since the analyzed morphological features do not account for process polarization but rather for branching level, then detecting these patterns is necessary to visualize these cells as a single cluster.

Grosso modo, the SCOP groups have a higher number of cells than the SS groups, which is already an indicator of a possible inflammatory process (Figure 4B; Tynan et al., 2010; De Biase and Bonci, 2019). More specifically, when segregating the cells into clusters, this hypothesis is reinforced by the increased number of cells in Clusters 0, 1, and 2, which resemble the “amoeboid” and “reactive” morphologies. Meanwhile, the SS groups predominantly appear in Clusters 4 and 5, which resemble the “homeostatic” morphology. Nevertheless, this research should be accompanied by omics studies to confirm these inferences.

Interestingly, despite the parallels between the two SCOP groups and the two SS groups, MorphoGlia reveals differences in both subregions concerning the strength of association with their clusters. For instance, in the SS groups, the Hilus microglia exhibited less morphological complexity and were more evenly distributed across the clusters, mirroring the SS-CA1 cluster association but with lower strength (Figure 4F). Similarly, the SCOP-Hilus group mirrored the SCOP-CA1 group but also showed a weaker association. This regional heterogeneity has been widely documented at both the genetic level (Ayata et al., 2018; Gosselin et al., 2014; Gosselin et al., 2017; Stratoulias et al., 2019; Tan et al., 2020) and the morphological level (Tynan et al., 2010; Colombo et al., 2022).

The MorphoGlia tool is user-friendly but still requires a certain level of computational expertise to accurately set optimal hyperparameters and interpret results. Additionally, code modifications may be necessary to customize and enhance the generated plots. While MorphoGlia effectively captures a wide range of microglial morphologies, the current pipeline relies on enzymatic immunohistochemistry, which requires manual preprocessing. To mitigate biases related to manual reconstruction during this process, implementing blinded conditions for sample identification is crucial. Additionally, establishing clearly defined criteria for image acquisition using the bright field microscope, along with thorough image preprocessing and final quality control, is essential not only for using MorphoGlia but for any morphological assessment. This method offers excellent visualization of microglial arborization but also necessitates manually connecting certain branches for optimal segmentation—a task that could potentially be automated in the future using neural networks.

One challenge in this analysis is the reliance on 2D images, which may hide some microglial processes. To address this limitation, immunofluorescence and confocal microscopy could be employed to streamline preprocessing and enhance automation. However, while these techniques could improve the clarity and automation of image processing, they might also increase image acquisition time and potentially reduce the visibility of microglial branches. Another issue arises from the use of 40x magnification in photomicrographs, which complicates the preservation of precise anatomical relationships. As a result, a manual cell count was conducted to assess the number and relative frequency of cells within specific clusters across different layers and lateral distances. In the future, using tiled images—offering greater structural context for image registration—could facilitate the automation of this step.

Building on this, spatial analysis of the CA1 region indicates that Cluster 0 cells are predominantly distributed within the SO and SR layers. Specific lateral locations and layers identified through MorphoGlia’s data highlight that Cluster 0 cells are abundant in the CA1 of the dorsal hippocampus, but particularly concentrated between 0.48 and 0.96 mm, while Cluster 1 cells show higher concentrations between 1.20 and 1.44 mm. Additionally, a significant abundance of Cluster 0 cells was observed in the 0.48 – 0.96 mm interval across all three sublayers of the hippocampal CA1. Moving forward, the objective is to automate this analysis and extend it to 3D imaging, enabling the creation of three-dimensional maps. Furthermore, integrating multiple elements within the same image will allow for more sophisticated spatial analyses between different components, such as examining the proximity of microglial clusters to neurons, blood vessels, amyloid-β plaques, and other structures. Additionally, comparing various AD models (e.g., scopolamine-induced, *3xTg-AD*, and *5xFAD*) by integrating their morphologies into a common data space would yield valuable insights into their distribution within the manifold and reveal shared and distinct morphological characteristics.

Another challenge is calculating the sample size required for effectively conducting spatial analysis, as clusters are identified using the density-based unsupervised algorithm HDBSCAN. A larger sample size would facilitate a more detailed analysis, and it is advisable for those interested in using MorphoGlia to consider this factor. Furthermore, once this tool is validated, the next step would involve automating the cellular counting process currently conducted manually. This approach would necessitate a regional template for the parcelation of brain regions. To achieve this, free access tools like Allen Brain Atlas could be utilized for automated spatial analyses.

Unlike neural networks that classify microglial morphology based on predefined labels, the MorphoGlia pipeline appears to display the full spectrum of microglial morphologies, avoiding the problem of *a priori* labeling, which can be difficult and subjective, even for experts. Instead, the approach used in MorphoGlia has the advantage of detecting subtle changes in the dataset. However, with large datasets, processing all cells would be computationally expensive. An interesting approach would be to use this pipeline to identify characteristic clusters in the study groups and then use these clusters to train neural networks. These neural networks could then identify the same clusters in new images. In this way, the use of neural networks *a posteriori* would help classify large datasets without requiring human training, as the images and unbiased classifications would already be available from MorphoGlia.

Finally, microglial morphology is not synonymous with microglial function; however, distinguishing these morphological states helps make inferences about their functionality (Paolicelli et al., 2022). Using our designed pipeline, MorphoGlia, we were able to create an unbiased classification that exhibited the spectrum of microglial morphological states and was biologically consistent with previously described literature. This approach allows for the identification of clusters within a spectrum, without the need for pre-assigned labels, which should later be evaluated using omics techniques. In this experiment, we found morphological states more associated with SCOP groups, consistent with reported findings, and states more associated with SS groups, also biologically consistent with literature reports. Furthermore, we were able to pinpoint the most affected spatial localization, identifying the SR as the layer with the highest cell count of pathology-associated clusters (Figure 5). In AD, this synapse-rich layer of the CA1 displays alterations in number of synapses and distribution of dendritic spines (Montero-Crespo et al., 2021), but also loss of integrity and atrophy that correlates with cognitive decline (Kerchner et al., 2012; Su et al., 2018). However, histological and omics approaches should be combined to gain a more comprehensive and accurate understanding of microglial status. Studies with spatial resolution could be particularly useful in confirming the inferences obtained through morphological classifications and spatial mappings, such as those offered by MorphoGlia.

Overall, this study demonstrates that MorphoGlia facilitates computational analysis for the scientific community while successfully capturing the continuous spectrum of microglial morphological states. It provides an unbiased classification that aligns with traditional descriptions in the literature, identifying clusters corresponding to well-known categories without predefined categorization. Moreover, MorphoGlia revealed for the first time morphological changes characterizing microglial morphologies within a SCOP-induced mouse model of AD. This differentiation highlights the tool’s capability to detect subtle morphological changes and distinguish between healthy and pathological conditions, as well as between subregions of the hippocampus. Additionally, the study identified the SR as the hippocampal layer that was most affected by SCOP treatment, with significant changes in microglial morphology correlating with cognitive decline in AD models.

MorphoGlia is designed to be an interactive and easy-to-use tool that utilizes Python, an open source programming language, therefore making it easily accessible to all users. Users are encouraged to directly modify the code to enhance and customize their research results.

In summary, MorphoGlia provides a comprehensive and nuanced understanding of microglial morphology, making it a valuable tool for studying microglial morphologies and gaining insights into tissue condition. These findings underscore the importance of combining morphological analysis with other techniques, such as omics studies, to obtain a more complete picture of microglial function and pathology.

## Data Availability

The raw data supporting the conclusions of this article will be made available by the authors, without undue reservation.

## Footnotes

1
https://pyinstaller.org/en/stable/

2
https://docs.python.org/3/library/tkinter.html

3
https://pypi.org/project/opencv-python/

4
https://pypi.org/project/scikit-learn/

5
https://pypi.org/project/umap-learn/

6
https://pypi.org/project/hdbscan/

7
https://connectivity.brain-map.org/3d-viewer?v=1
