# Supplementary material for: MorphoGlia, an interactive method to identify and map microglia morphologies, demonstrates differences in hippocampal subregions of an Alzheimer’s disease mouse model
Source: Front Cell Neurosci. 2024 Dec 3;18:1505048. doi: 10.3389/fncel.2024.1505048 (PMC11653188; doi:10.3389/fncel.2024.1505048)
Supplement: Supplementary file 1 [file Table_1.DOCX]

Supplementary Material

|  | **Cluster 0** | **Cluster 1** | **Cluster 2** | **Cluster 3** | **Cluster 4** | **Total** |
| --- | --- | --- | --- | --- | --- | --- |
| **SS CA1** | 27  (9.54%) | 30  (10.60%) | 53  (18.73%) | 95  (33.57%) | 78  (28.33%) | 283  (100%) |
| **SCOP CA1** | 129  (41.35%) | 49  (15.71%) | 60  (19.23%) | 53  (16.99%) | 21  (6.73%) | 312  (100%) |
| **SS Hilus** | 73  (26.45%) | 25  (9.06%) | 59  (21.38%) | 67  (24.28%) | 52  (18.84%) | 276  (100%) |
| **SCOP Hilus** | 126  (36.31%) | 53  (15.27%) | 70  (20.17%) | 72  (20.75%) | 26  (7.49%) | 347  (100%) |
| **Total** | 355 | 157 | 242 | 287 | 177 | 1,218 |

**Supplementary Table 1.** Contingency Table of Study Groups and Clusters. The table shows the frequencies and percentages of each cluster in each study group.

| **Type of analysis** | **Parameter** | **ID** |
| --- | --- | --- |
| Soma | Area | So1 |
| Soma | Perimeter | So2 |
| Soma | Circularity | So3 |
| Soma | Compactness | So4 |
| Soma | Orientation | So5 |
| Soma | Feret diameter | So6 |
| Soma | Eccentricity | So7 |
| Soma | Aspect Ratio | So8 |
| Skeleton | Endpoints | Sk1 |
| Skeleton | Junctions | Sk2 |
| Skeleton | Branches | Sk3 |
| Skeleton | Initial points | Sk4 |
| Skeleton | Total branches length | Sk5 |
| Skeleton | Ratio branches | Sk6 |
| Fractal | Convex Hull area | F1 |
| Fractal | Convex Hull perimeter | F2 |
| Fractal | Convex Hull compactness | F3 |
| Fractal | Convex Hull eccentricity | F4 |
| Fractal | Fractal dimension | F5 |
| Fractal | Covex Hull feret diameter | F6 |
| Cell | Area | C1 |
| Cell | Perimeter | C2 |
| Cell | Circularity | C3 |
| Cell | Compactness | C4 |
| Cell | Feret diameter | C5 |
| Cell | Eccentricity | C6 |
| Cell | Aspect ratio | C7 |
| Cell | Cell solidity | C8 |
| Cell | Cell convexity | C9 |
| Sholl | Max distance | Sh1 |
| Sholl | Crossing processes | Sh2 |
| Sholl | Circles | Sh3 |

**Supplementary Table 2.** Parameters assessed for Recursive Feature Elimination in MorphoGlia. The table shows the classic morphometric features of each type of analysis computed by MorphoGlia and the identification used for correlation plot.


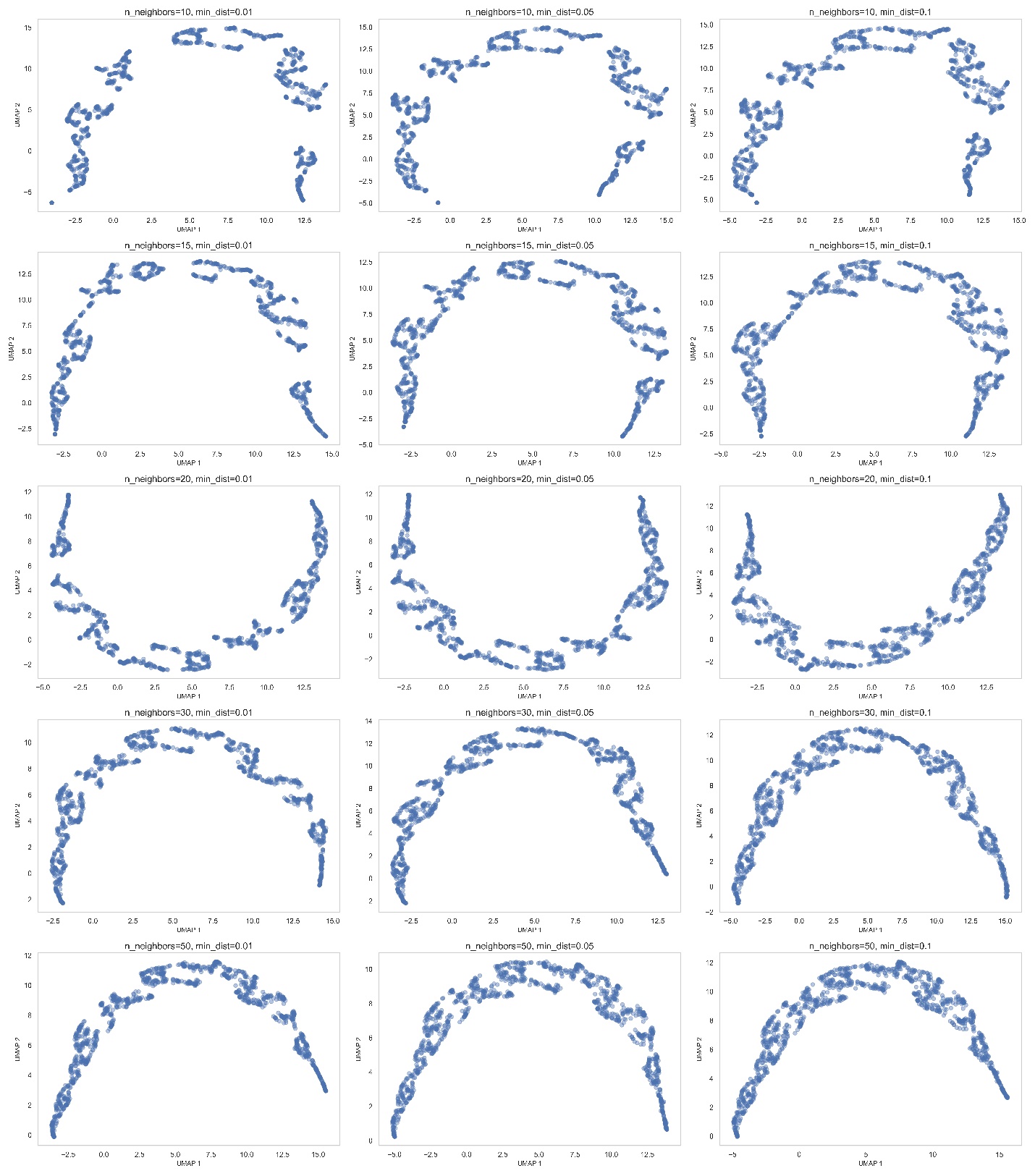


**Supplementary Figure 1.** UMAP Grid Test. To explore local and global structures of the data, assess robustness, and identify the optimal hyperparameters, we performed a grid test. This analysis provides insights into the manifold structure. Notably, despite varying hyperparameters, the resulting structure consistently exhibits a curved spectrum, demonstrating the stability and resilience of the data's intrinsic structure.


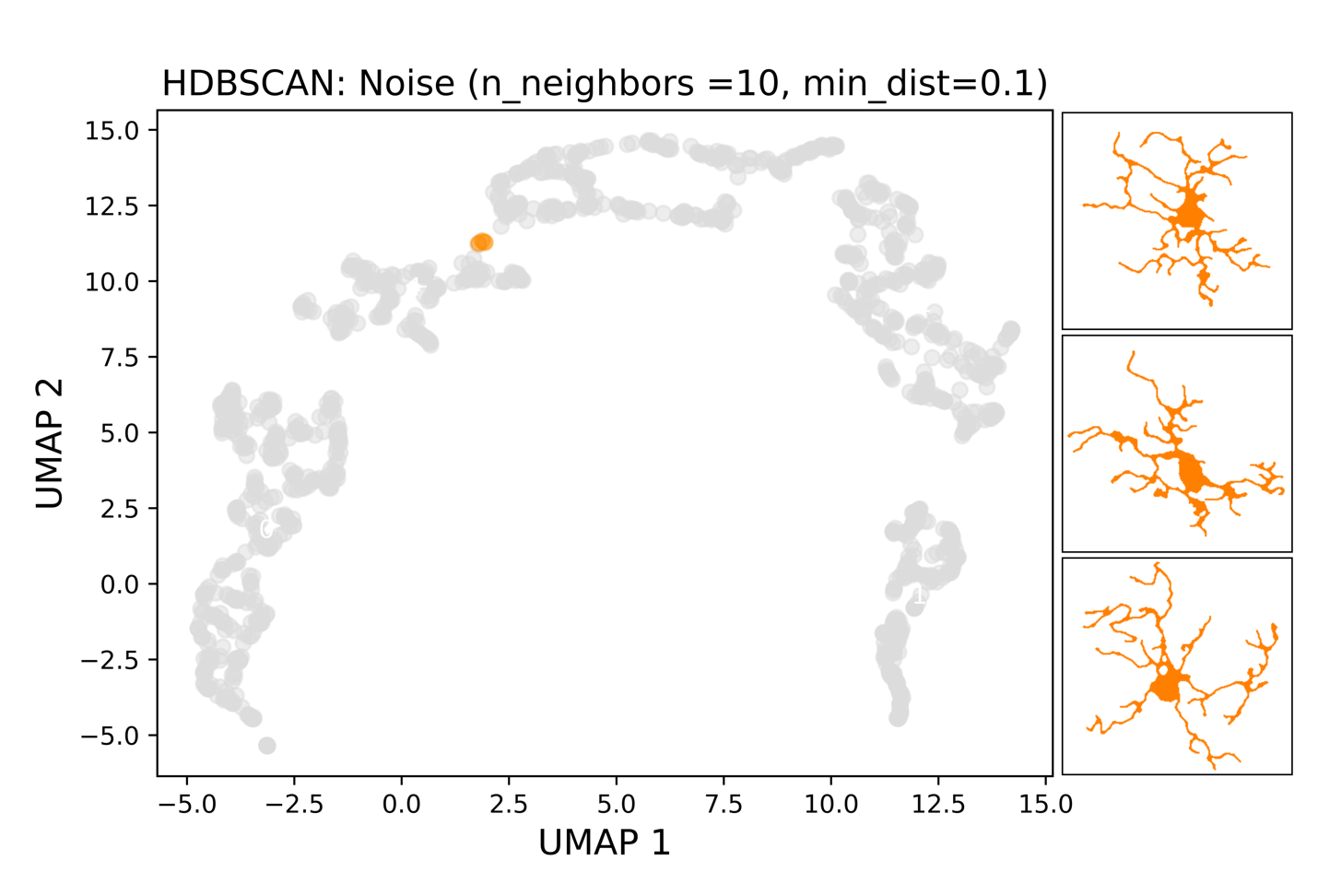


**Supplementary Figure 2.** Noise detection by HDBSCAN algorithm. The Hierarchical Density-Based Spatial Clustering of Applications with Noise (HDBSCAN) algorithm operates with minimal assumptions about cluster formation. It identifies clusters as regions of high density distinctly separated by low-density areas, eliminating the need to predefine the number of clusters. This functionality allows HDBSCAN to effectively manage data noise by excluding points outside high-density regions, thereby enhancing its robustness. This capability facilitates the detection of outliers or preprocessing errors. However, in this case, there seems to be no preprocessing error in the cell data. Accordingly, it may be inferred that the detected noise represents transitional morphological states between two clusters, which do not conform to any high-density regions. Further investigation is warranted to clarify this issue. The orange marker indicates the location of three data points represented as noise by HDBSCAN within the structure identified by UMAP. On the left, the corresponding three cells are displayed for visualization.


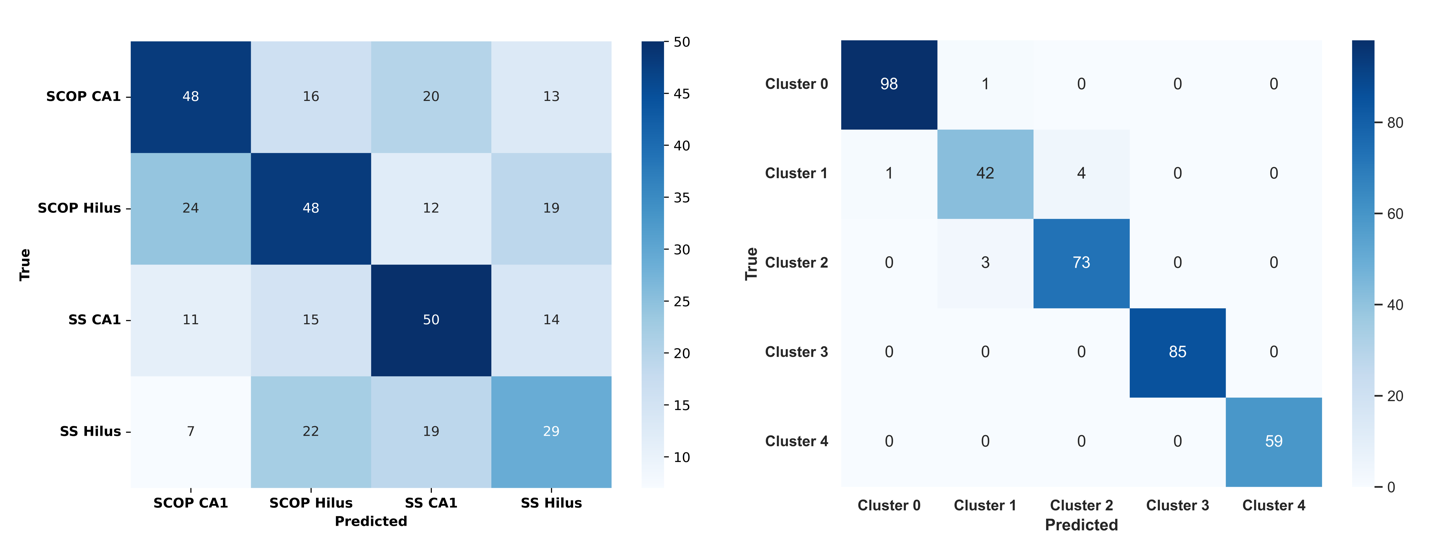


**Supplementary Figure 3.** Confusion Matrices of the Study Groups and MorphoGlia Clusters. Contrasting the confusion matrices generated for distinguishing the study groups and MorphoGlia clusters reveals significant differences in classification accuracy. When evaluating the study groups directly, the accuracy is 0.47, indicating considerable overlap in morphological states. In contrast, clustering with the MorphoGlia pipeline significantly improves classification accuracy to 0.97. This improvement suggests that there is a mixing of morphological states within the study groups, akin to Simpson's Paradox, and that the MorphoGlia pipeline effectively unveils distinct morphological clusters.


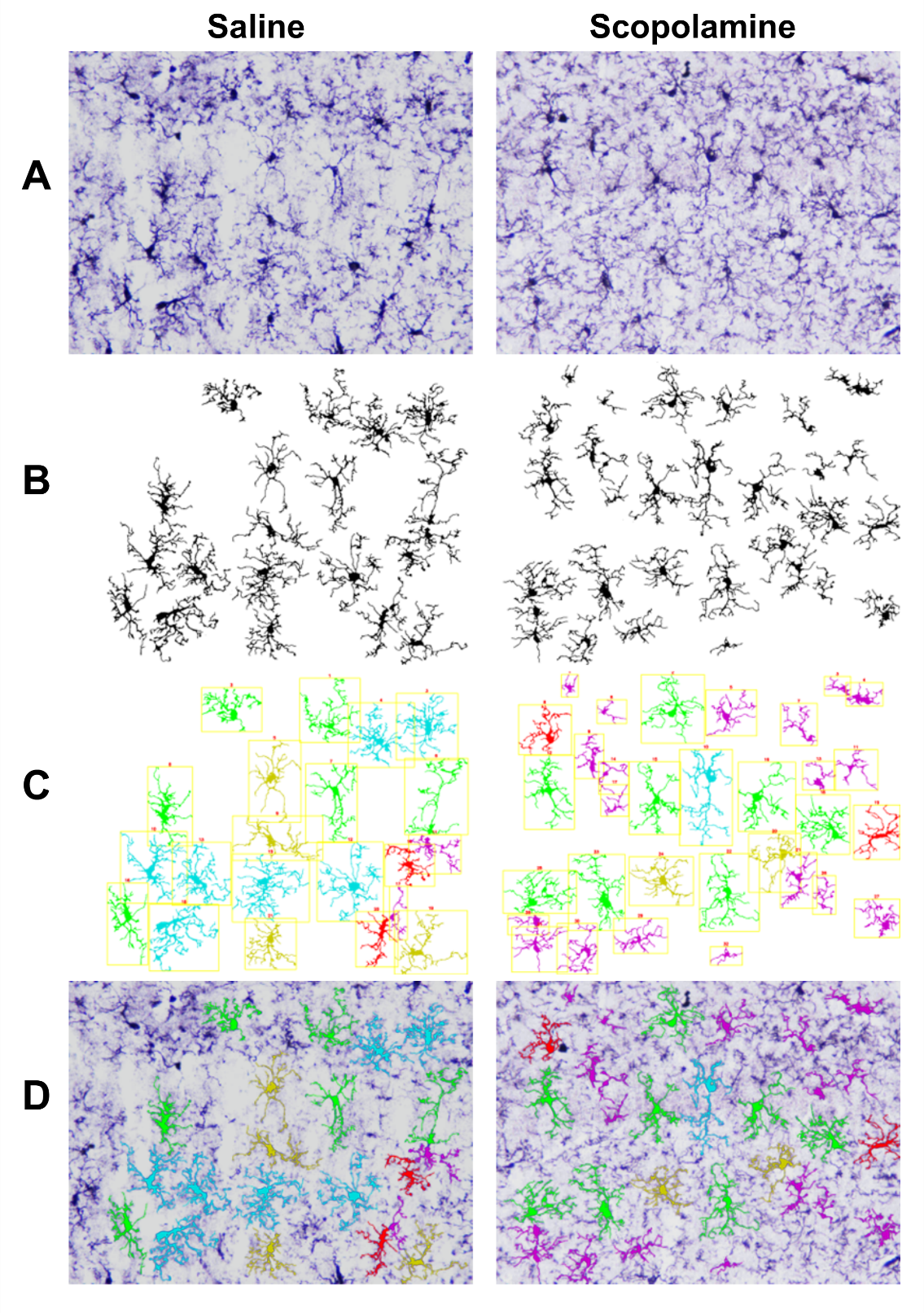


**Supplementary Figure 4.** Comparison of processing between saline and scopolamine groups. A) Raw Image: Image acquired by the microscope; B) Binary Image (input image): Following the application of hybrid preprocessing techniques, including filters and manual refinement, the resulting binary image serves as the input for the MorphoGlia pipeline; C) Spatial Visualization of the Clustered Cells (output image): Image displaying color-coded cells according to their cluster in their spatial arrangement; D) Overlapping Images: Spatially clustered cells can be overlapped onto the raw images to facilitate spatial analysis.


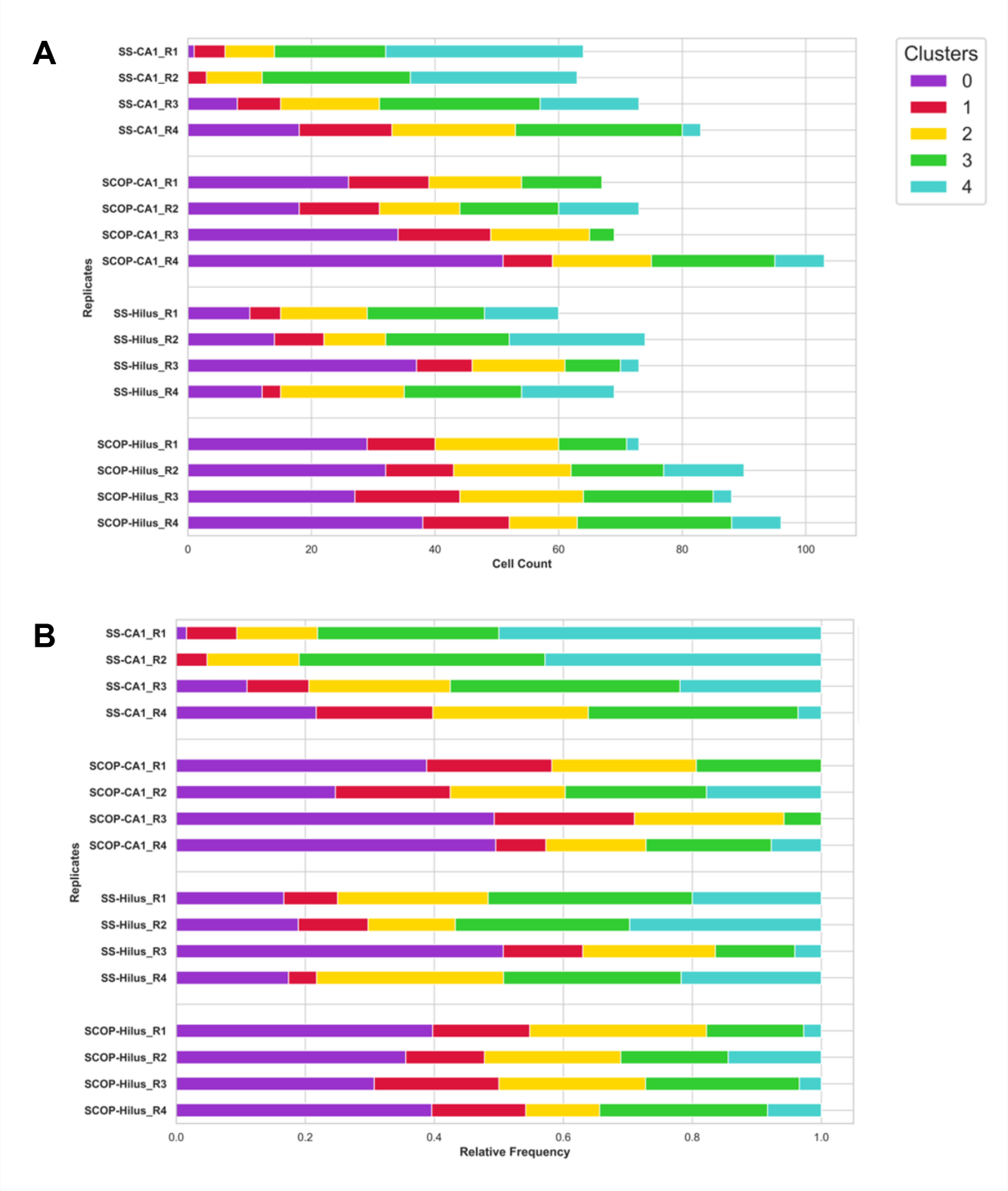


**Supplementary Figure 5.** Clustered cells count and relative frequency by replicate. A) Cell count of clusters by replicate; B) Relative frequency of clusters by replicate.
